# Statistical optimisation of polyhydroxyalkanoate production in *Bacillus endophyticus* using sucrose as sole source of carbon

**DOI:** 10.1007/s00203-021-02554-6

**Published:** 2021-09-22

**Authors:** M. Geethu, H. Raghu Chandrashekar, M. S. Divyashree

**Affiliations:** 1 Department of Biotechnology, Manipal Institute of Technology, Manipal Academy of Higher Education, Manipal, Udupi, Karnataka 576104 India; 2 Department of Pharmaceutical Biotechnology, Manipal College of Pharmaceutical Sciences, Manipal Academy of Higher Education, Manipal, Udupi, Karnataka 576104 India

**Keywords:** *Bacillus endophyticus*, Biopolymer, Bioreactor, Polyhydroxyalkanoate (PHA), Shake flask cultivation

## Abstract

Microorganisms have been contemplated as a promising source for the inexhaustible synthesis of many novel materials utilizing renewable sources. Among many of such products, polyhydroxyalkanoate (PHA) remains as an essential biodegradable polymer with functions similar to conventional plastics. *Bacillus endophyticus* is capable of accumulating biopolymer PHA in nutrient limiting conditions with excess of carbon source. Screening and optimizing the parameters for increased PHA production was done statistically. The optimized medium gave a maximum yield of 46.57% which was in well agreement with the given predicted value provided by response surface methodology model yield of 47.02%. Optimal media conditions when extrapolated in bioreactor gave an even higher production percentage of 49.9. This is the first report highlighting 49% of polyhydroxybutyrate statistically using sucrose as a source. The main highlight of the study was the use of wild type strain for producing high quality PHA using simple carbon source which can be a starting platform for using this strain for large scale PHA production industrially. FTIR and ^1^HNMR analysis confirmed the polymer produced.

## Introduction

Worldwide interest to use bio-based polymers has accelerated as an eco-friendly alternative which resembles plastic in their physio-chemical properties to overcome the increased demands in the rapidly developing fields like biomedical and industrial sector. Total demand for the biodegradable polymers in North America, Western Europe and Asia reached around 85,000 metric tons and the estimated consumption of biodegradable polymers has increased in these years (Akaraonye et al. 2010). Microorganisms are considered as a promising source for the inexhaustible synthesis of many novel materials utilizing renewable sources. Many eco-friendly materials have been produced as reserve molecules under stress which can be used to resolve the putative problems with several industrial applications. Among many of such products, polyhydroxyalkanoates (PHAs) remain as an essential biodegradable polymer with functions similar to conventional plastics (Amache et al. 2013). A sequence of complex stages starting from biomass accumulation, polymer synthesis and extraction of polymer to recovery is associated with PHA production which significantly influences PHA quality (Mohammed et al. 2019). In recent years, an inclination towards efficient wild strain isolation and screening with quality PHA accumulation capable of utilizing unconventional substrates and altered metabolic pathways was explored widely.

PHA accumulation and bioprocess optimization is very significant to ensure the quality and quantity of biopolymer synthesized via wild type strains. Industries mainly relay on strains like *Cupriavidus necator* and *Alcaligenes latus* due to their high production rate and PHA yield. In strains like *Corynebacterium, Nocardia* and *Rhodococcus* are the only wild type strains reported to synthesize commercially important copolymers from simple carbon sources like glucose, which allowed a decrease in the production cost of the polymer (Kaur and Roy 2015). Decreased yield of the polymer significantly contribute to the overall economy of the polymer as less bioconversion require increased substrate availability to uplift the PHA production (Prados and Maicas 2016). This leads to increase in the overall production rate and to avoid this rapid improvement in the economics of PHA production process need to be implemented by incorporating significant process designing and utilization of cheap substrate thereby increasing PHA yield. Optimization of the bacterial requirement for maximal PHA production enabled by incorporating cheap readily available substrates in Gram positive stains has witnessed a trend recently (Chen and Jiang 2018).

PHA production by microorganism mainly depends on the carbon source and other nutrients provided. A strategy can be introduced to induce pressure on PHA producers to convert available carbon source to biopolymer. This condition can be achieved by optimising the ecosystem and by maintaining a feast and famine condition to organism (Aramvash et al. 2015). As conventional method of optimization consumes lot of time and the relative interactions of different parameters cannot be readily studied, statistical experimental design can be employed to avoid the limitations of one factor at a time method (Tripathi et al. 2013; Din et al. 2014). Screening of significantly influencing parameters and analysing the interaction between them enable to depict their combined effect through factorial design followed by response surface methodology (RSM). Statistical methods of optimisation remains an important strategy in achieving optimal PHA concentration before stepping into large scale production in the presence of cheap carbon sources (Kaur and Roy 2015).

This work mainly focuses on providing experimental insight towards hypothesis linking PHA accumulation and the pattern of utilising sucrose as a major carbon source. Statistical optimization of various production parameters during PHA biosynthesis in *Bacillus endophyticus*, utilizing sucrose as a sole source of carbon was carried out. Plackett–Burman design (PBD) and central composite design (CCD) were predominantly used in optimising and screening the most influencing parameter in increasing PHA production. The correlation of the increase in cell density and PHA production was also evaluated during this study. The novelty of the work lies in the higher accumulation of PHA by wild type *B. endophyticus* strain by utilising simple carbon source for polymer biosynthesis.

## Materials and methods

### Microorganism and culture maintenance

*Bacillus endophyticus,* organism of interest was previously isolated and confirmed in the lab and was frequently sub-cultured on nutrient agar medium (Himedia, Mumbai, India) and stored at 4 °C. Commonly used staining methods were performed to confirm the microorganism. Primary staining methods like gram staining and endospore staining were performed to confirm *Bacillus* spp.

### Screening and optimization of PHA production media components using statistical experimental design

#### Plackett–Burman design (PBD)

PBD are predominantly used to screen the factors in an experiment. It is necessary to optimize the production medium for PHA biosynthesis by *B. endophyticus* using sucrose as major carbon source. PB design is largely used to detect the main effects economically and helps in estimating the variances of the factors using minimum experiments. 95% of relative significance level was chosen to carry out the experimental trials with five factors by experiments designed by Minitab 17. High (+ 1) and Low (− 1) level were coded by PBD and real values of the independent variables were represented in Table 1.

**Table 1:** Different levels of experimental factors selected for PHA production *by Bacillus endophyticus* using Plackett–Burman design

No	Components (factors)	Low level (− 1) g/L	High level (+ 1) g/L
1	Na_2_HPO_4_	0.5	5
2	KH_2_PO_4_	1.5	15
3	(NH_4_)_2_SO_4_	1.5	15
4	MgSO_4_·7H_2_O	0.2	2
5	Sucrose	20	40

Biopolymer production was done in cotton plugged 250 mL Erlenmeyer flask with 50 mL working volume of production medium (PM) which was varied based on the composition obtained by Minitab software which included (NH4)_2_SO_4_, KH_2_PO_4_, Na_2_HPO_4_ 2H_2_O, MgSO_4_7H_2_O Sucrose. All the shake flask experiment was carried out in duplicates. The pH of the PM was maintained at 7 before inoculation. 10% of the inoculum was added to production medium which was later incubated at 32 °C for 72 h.

#### Central composite design (CCD)

Central composite design was carried out for PHA optimisation studies using sucrose as carbon source. Different combinations of three significant variables (Na_2_HPO_4_, KH_2_PO_4_ and sucrose) found to influence PHA production by PBD was used in CCD. CCD with three variables at 5 levels (− 2, − 1, 0, + 1, + 2) with 20 experiments was used to fit the second order polynomial model. The study was performed to understand the influence of these parameters on responses like biomass and PHA production. The variables and levels for CCD were represented in Table 2. The parameters for the study were selected on literature which was used as components for PHA production medium (Mohammed et al. 2019).

**Table 2:** Low and high levels for 3 factors screened for PHA yield by *B. endophyticus*

No	Components (factors)	Units	Low level (− 1)	High level (+ 1)
1	Na_2_HPO_4_ (g/L)	g/L	1	2
2	KH_2_PO_4_ (g/L)	g/L	1	2
3	Sucrose (g/L)	g/L	20	60

### Bioreactor studies

The initiation of PHA synthesis and the maximal production can be represented as time course study in bioreactor. Batch fermentation to evaluate initiation of PHA biosynthesis was studied in a 3 L bioreactor (Bioflo 110-New Brunswick scientific). The optimised basal medium obtained after statistical optimisation was ran in 3 L bioreactor. The inoculum (10% for I L of PM) was cultured until 24 h at 32 °C with 150 rpm were aseptically transferred to 3 L of production medium aseptically. Dissolved oxygen and pH values were pre-set and regulated by corresponding probes attached to bioreactor system. 200 rpm was the agitation rate maintained and addition of 0.1 N polyethylene glycol was carried out occasionally as antifoaming agent during foaming. Samples (15 mL) were drawn at regular intervals to study biomass and PHA production rate of *B. endophyticus*.

### Estimation of cell dry mass

The culture broth was collected and centrifuged at 10,000 rpm (Plastocraft SSR-V/FM) for 10 min and the supernatant was analyzed for residual sugar by dinitrosalicylic acid method (Miller 1959) through spectrophotometric analysis at 540 nm. The harvested cell pellet was washed with distilled water and then dried at 80 °C to a constant weight. As it was gram positive strain, the chances of cell wall disruption was found to be negligible even after exposing to temperature as high as 80 °C (Russell 2003).

### Extraction of PHA

The PHA content of the cells was estimated by subjecting dry cells into hydrolysis for 1 h using 4% Sodium hypochlorite solution at 40 °C (Slepecky and Law 1960) followed with water and acetone wash for the centrifuged hydrolysate the remained residue obtained after wash was dissolved in solvent chloroform. Gravimetric quantification of obtained PHA film was done routinely after each experimental trial (Lakshman and Shamala 2006).

### ***FTIR and ***^***1***^***HNMR analysis of PHA***

The PHA film obtained after dissolving in chloroform was pelletized using potassium bromide (KBr). FTIR spectrum was recorded for each pelletized sample in the sample chamber of FTIR spectrophotometer and was exposed to infra-red radiation. The obtained spectrum using Shimadzu 8400 S was in the spectral range of 4000–400 cm^−1^*.* 5 mg of PHA film was used for ^1^H NMR and characterized in Bruker Ascend 400 NMR spectrometer at 22 °C.

## Results

### Sequential statistical optimisation of production medium

#### Plackett–Burman design

**Fig. 1:**
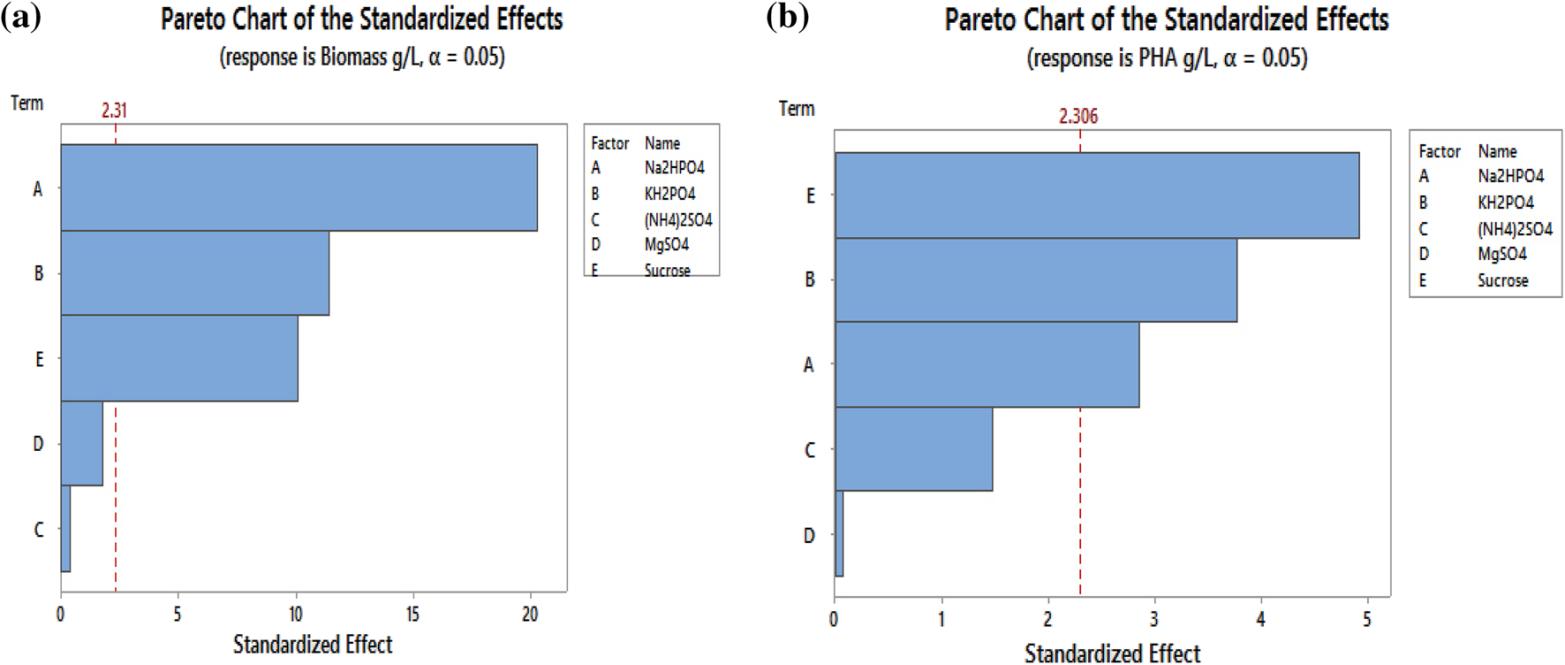
Standardized effects on the fermentative parameters shown in Pareto chart for **a** biomass (g/L) and **b** PHA (g/L) production by *B. endophyticus*

Software driven experimental trial was developed (Table 3) using Minitab 17 and experiments were carried out sequentially. The main aim of this screening study was to identify the parameters that were highly significant during PHA production. The responses, Biomass and PHA yield was represented in g/L and the relative significance of five factors (Na_2_HPO_4_, KH_2_PO_4_, (NH_4_)_2_SO_4,_ MgSO_4_·7H_2_O and concentration of sucrose) at two levels (low and high). The significance level was 95% and 12 experiments with 3 centre points were carried out in shake flask. The experimental and predicted values of biomass and PHA are represented in Table 3. Statistical analysis of PB design on biomass (g/L) and PHA production (g/L) was represented in Tables 4 and 5 respectively as ANOVA table and the effects were represented in Pareto chart for the same in Fig. 1 (a and b).

**Table 3:** Experimental model of Plackett–Burman design on biomass and PHA yield by *B. endophyticus* using sucrose as carbon source

Run	Na_2_HPO_4_ (g/L)	KH_2_PO_4_ (g/L)	(NH_4_)SO_4_	MgSO_4_·7H_2_O	Sucrose g/L	Biomass (g/L)		PHA (g/L)	
						Experimental	Predicted	Experimental	Predicted
1	5.00	1.50	15.00	0.2	20	2.130	2.21033	0.220	0.21533
2	5.00	15.00	1.50	2.0	20	3.028	2.98367	0.662	0.84133
3	0.50	15.00	15.00	0.2	40	2.558	2.31233	0.962	0.90200
4	5.00	1.50	15.00	2.0	20	2.206	2.06100	0.266	0.22400
5	5.00	15.00	1.50	2.0	40	3.894	3.83100	1.766	1.42000
6	5.00	15.00	15.00	0.2	40	3.900	4.01500	1.186	1.23800
7	0.50	15.00	15.00	2.0	20	1.200	1.31567	0.316	0.33200
8	0.50	1.50	15.00	2.0	40	1.126	1.20567	0.428	0.46667
9	0.50	1.50	1.50	2.0	40	1.114	1.17100	0.486	0.64000
10	5.00	1.50	1.50	0.2	40	2.966	3.02300	0.806	0.96733
11	0.50	15.00	1.50	0.2	20	1.308	1.43033	0.338	0.49667
12	0.50	1.50	1.50	0.2	20	0.602	0.47300	0.360	0.05267
13	2.75	8.25	8.25	1.1	30	2.858	2.90933	1.694	1.68267
14	2.75	8.25	8.25	1.1	30	2.940	2.90933	1.698	1.68267
15	2.75	8.25	8.25	1.1	30	2.930	2.90933	1.656	1.68267

**Table 4:** Statistical analysis of Plackett–Burman design on biomass yield with five fermentative parameters

Source	*df*	Adj SS	Adj MS	*F* value	*p* value	Main effect	Std. error
Model	6	14.9854	2.49756	118.35	0		
Linear	5	13.6711	2.73422	129.57	0		
Na_2_HPO_4_	1	8.6972	8.69722	412.14	0	1.7027	0.0419
KH_2_PO_4_	1	2.7495	2.74946	130.29	0	0.9573	0.0419
(NH_4_)_2_SO_4_	1	0.0036	0.00361	0.17	0.69	0.0347	0.0419
MgSO_4_·7H_2_O	1	0.0669	0.0669	3.17	0.113	− 0.1493	0.0419
Sucrose	1	2.1539	2.15392	102.07	0	0.8473	0.0419
Curvature	1	1.3142	1.31424	62.28	0		
Error	8	0.1688	0.0211				
Lack-of-fit	6	0.1648	0.02747	13.73	0.069		
Pure error	2	0.004	0.002				
Total	14	15.1542					

*df* degrees of freedom, *SS* sum of squares

*S* = 0.14526; *R*^*2*^ = 98.89%; *R*^*2*^_(adj.)_ = 98.05%; *F* = MS_(Factor)_ /MS_(Error)_

**Table 5:** Statistical analysis of Plackett–Burman design on PHA yield with five fermentative parameters

Source	*df*	Adj SS	Adj MS	*F* value	*p* value	Main effect	Std. error
Model	6	4.58603	0.76434	18.41	0		
Linear	5	2.02502	0.405	9.75	0.003		
Na_2_HPO_4_	1	0.33869	0.33869	8.16	0.021	0.3360	0.0588
KH_2_PO_4_	1	0.59141	0.59141	14.24	0.005	0.4440	0.0588
(NH_4_)_2_SO_4_	1	0.09013	0.09013	2.17	0.179	− 0.1733	0.0588
MgSO_4_·7H_2_O	1	0.00023	0.00023	0.01	0.943	0.0087	0.0588
Sucrose	1	1.00457	1.00457	24.19	0.001	0.5787	0.0588
Curvature	1	2.56101	2.56101	61.68	0		
Error	8	0.33217	0.04152				
Lack-of-fit	6	0.33109	0.05518	102.7	0.01		
Pure error	2	0.00107	0.00054				
Total	14	4.9182

**Fig. 2:**
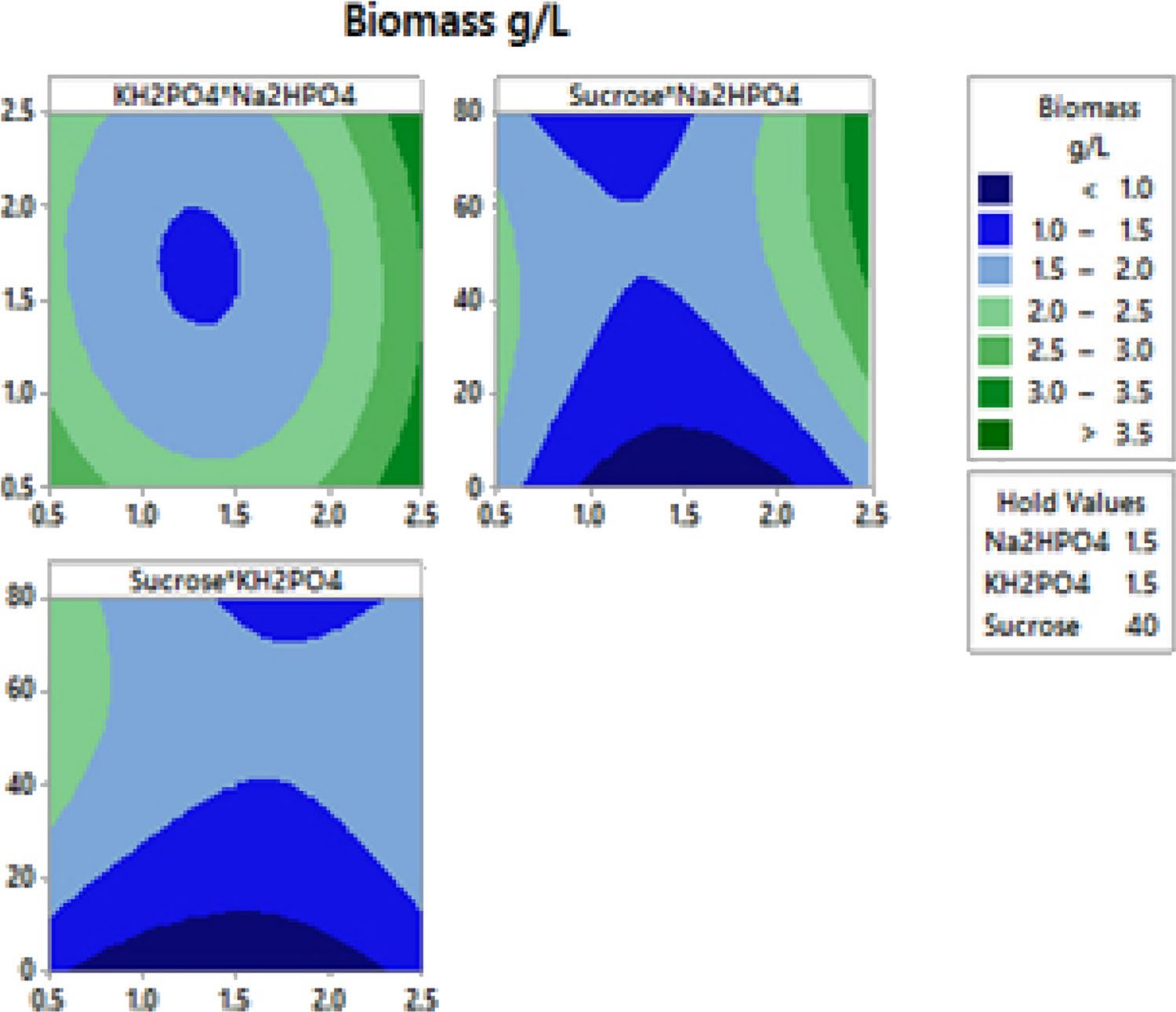
2D contour plot showing the interactive effects of three variables showing the interaction effect of Biomass g/L of *B.endophyticus* using sucrose as carbon source

The graphical representation of this phenomenon is given in Pareto chart Fig. 2a, b, which details the relevance of main effect to determine how far these factors are different from zero. The horizontal column in the graph represents the value for 95% confidence level and gave a *T* value of 2.18 as a vertical line in the plot. The *T* value indicates the minimum statistically significant effect for 95% significant level. From Pareto chart Fig. 1a, b, it was observed that the concentration of three factors (Na_2_HPO_4_, KH_2_PO_4_ and concentration of sucrose) was positively affecting biomass and PHA yield. Independent variables above 95% were considered as significantly influencing biomass and PHA yield.

Designing the screening experiment was to evaluate the factors that highly influence PHA production. Before specific screening of the highly influencial parameters on the production, Plackett–Burman studies were carried out which gave an idea on which the production may relay. The factors used for the experiments were selected that had close link with the production during shake flask cultivation done prioirly. This was clearly visualised in pareto chart which showed the standardised lineance of the production towards each factors. The cumulative inference is that the concentration of Na_2_HPO_4_, KH_2_PO_4_ and sucrose influences the overall yield of biomass and PHA production during the course of growth. Sucrose concentration readily had an influence on biomass and the production of biopolymer. During biomass increment, the role of KH_2_PO_4_and Na_2_HPO_4_ was found to be significant which indicates the trivial role of potassium and sodium ions for different transport system and cytoplasmic signalling (Wang and Bakken 1998; Bora 2013). This enable cells to divide and increase in cell mass. Whereas during PHA production, the role of (NH_4_)_2_SO_4_ was found to be insignificant which justifies the correlation of limited nitrogen source for enhancing PHA production.

Regression equation for the Model (Biomass g/L):

$$\begin{aligned} {\text{Biomass}}\;{\text{g}}/{\text{L }} & = - 0.{657 } + \, 0.{\text{3784 Na}}_{{2}} {\text{HPO}}_{{4}} \, + \, 0.0{7}0{\text{91 KH}}_{{2}} {\text{PO}}_{{4}} \\ &\quad + \, 0.00{257 }\left( {{\text{NH}}_{{4}} } \right){\text{2SO}}_{{4}} - 0.0{83}0{\text{ MgSO}}_{{4}} \\&\quad + \, 0.0{\text{4237 Sucrose }} + \, 0.{74}00{\text{ Ct Pt}}{.} \\ \end{aligned}$$

Regression equation for the Model (PHA g/L):

$$\begin{aligned} {\text{PHA}}\;{\text{g}}/{\text{L }} & = \, - 0.{594 } + \, 0.0{\text{747 Na}}_{{2}} {\text{HPO}}_{{4}} + \, 0.0{\text{3289 KH}}_{{2}} {\text{PO}}_{{4}} \\ & \quad - \, 0.0{1284 }\left( {{\text{NH}}_{{4}} } \right)_{{2}} {\text{SO}}_{{4}} + \, 0.00{\text{48 MgSO}}_{{4}} \\ & \quad + \, 0.0{\text{2893 Sucrose }} + { 1}.0{\text{33 Ct Pt}}{.} \\ \end{aligned}$$

Effect of carbon source, sucrose concentration (0–4%) on PHA biosynthesis were studied in *B. endophyticus* in PHA production medium. The correlation of biomass and PHA in the excess presence of sucrose was studied as batch cultivation using statical optimisation tool. The biomass accumulation is very essential, as there is a direct relationship between the biomass and PHA content as PHA is directly accumulated within the cytoplasm. Residual biomass is the catalytic component responsible for the metabolic activity, so increase in the biomass determine the quantity of PHA that was produced potentially. So the cell growth balance is necessary during the growth phase to avoid incomplete PHA accumulation. High biomass % is directly proportional to PHA concentration so it is necessary to decide the harvesting time to attain maximum PHA production (Pijuan et al. 2009). Commercial production of PHA relay mainly on fed batch and batch cultivation. Taking this into consideration, batch cultivation in shake flask was carried out and the optimal concentration of components obtained via statistical optimisation was extrapolated in bioreactor. It was necessary to carry out RSM which helped in specifically determine the second order behaviour of the parameters (Chen and Jiang 2018). It also enables the correlation between the factors and responses with the best combination of the parameters can be used by the model to obtain maximum yield. Using factorial experimental design, the errors generated gets nullified and the overall effects of variable provides mutual interaction of the parameters through factorial experimental design. The maximum PHB yield was 49.47% of biomass after 72 h of cultivation which was not reported by any *Bacillus* strain so far utilising sucrose as sole carbon source (Mohammed et al. 2019).

### Central composite design and response surface methodology

Central composite design was carried out for PHA optimisation studies using sucrose as carbon source. Different combinations of three significant variables (Na_2_HPO_4_, KH_2_PO_4_ and sucrose) found to influence PHA production by PBD were used in CCD. CCD with three variables at five levels (− 2, − 1, 0, + 1, + 2) with 20 experiments was used to fit the second order polynomial model. The study was performed to understand the influence of these parameters on responses like biomass and PHA production. The variables and levels for CCD were represented in Table 6 and the design table obtained by Minitab 17 is represented in Table 7.

$$\begin{aligned} {\text{Biomass}}\;{\text{g}}/{\text{L}}\, & = \,4.573 \, - \, 3.541Z_{1} - \, 1.729 \, Z_{2} + \, 0.02468 \, Z_{3} \\ & \quad + \, 1.0907 \, Z_{1} \times \, Z_{1} + \, 0.5217 \, Z_{2} \times \, Z_{2} \\ & \quad - \, 0.000296 \, Z_{3} \times \, Z_{3} + \, 0.140 \, Z_{1} \times \, Z_{2} \\ & \quad + \, 0.01140 \, Z_{1} \times \, Z_{3} - \, 0.00510 \, Z_{2} \times \, Z_{3} . \\ \end{aligned}$$

$$\begin{aligned} {\text{PHA}}\;{\text{g}}/{\text{L}} & = 1.754 \, - \, 1.003 \, Z_{1} - \, 1.137 \, Z_{2} + \, 0.01335 \, Z_{3} \\ & \quad + \, 0.1945 \, Z_{1} \times Z_{1} + \, 0.1835 \, Z_{2} \times Z_{2} \\ & \quad - 0.000237 \, Z_{3} \times Z_{3} + \, 0.288 \, Z_{1} \times Z_{2} \\ & \quad + \, 0.00400 \, Z_{1} \times Z_{3} + \, 0.00205 \, Z_{2} \times Z_{3} . \\ \end{aligned}$$

**Table 6:** Central composite experimental design for optimising three parameters

Run	Na_2_HPO_4_ (g/L)		KH_2_PO_4_ (g/L)		Sucrose		Biomass (g/L)		PHA (g/L)		PHA (%)
	Coded value	Real value	Coded value	Real value	Coded value	Real value	Experimental	Predicted	Experimental	Predicted	
1	− 1	1.0	− 1	1.0	− 1	20	1.73	1.70	0.54	0.58	31.21
2	1	2.0	1	2.0	− 1	20	1.89	1.81	0.61	0.56	32.27
3	1	2.0	− 1	1.0	1	60	2.59	2.54	0.74	0.71	28.57
4	− 1	1.0	1	2.0	1	60	1.58	1.66	0.41	0.43	25.94
5	0	1.5	0	1.5	0	40	1.54	1.64	0.55	0.57	35.71
6	0	1.5	0	1.5	0	40	1.66	1.64	0.57	0.57	34.34
7	1	2.0	− 1	1.0	− 1	20	1.91	1.84	0.56	0.57	29.30
8	− 1	1.0	1	2.0	− 1	20	1.56	1.61	0.31	0.37	19.87
9	− 1	1.0	− 1	1.0	1	60	1.95	2.03	0.56	0.64	28.71
10	1	2.0	1	2.0	1	60	2.36	2.40	0.88	0.86	37.28
11	0	1.5	0	1.5	0	40	1.72	1.68	0.80	0.61	46.51
12	0	1.5	0	1.5	0	40	1.74	1.68	0.55	0.61	31.6
13	− 2	0.5	0	1.5	0	40	1.96	1.87	0.60	0.52	30.61
14	2	2.5	0	1.5	0	40	2.58	2.67	0.81	0.87	31.39
15	0	1.5	− 2	0.5	0	40	1.82	1.86	0.79	0.75	43.4
16	0	1.5	2	2.5	0	40	1.58	1.54	0.60	0.61	37.97
17	0	1.5	0	1.5	− 2	0	0.22	0.29	0.00	− 0.02	Nil
18	0	1.5	0	1.5	2	80	1.20	1.12	0.27	0.26	22.5
19	0	1.5	0	1.5	0	40	1.18	1.18	0.46	0.50	39.31
20	0	1.5	0	1.5	0	40	1.17	1.18	0.46	0.50	39.31

**Table 7:** Analysis of variance for biomass (g/L) with three factors

Source	*df*	Adj SS	Adj MS	*F* value	*p* value
Model	11	5.23547	0.47595	51.75	0
Blocks	2	1.02819	0.5141	55.9	0
Linear	3	1.42927	0.47642	51.8	0
Z_1_	1	0.62885	0.62885	68.37	0
Z_2_	1	0.09986	0.09986	10.86	0.011
Z_3_	1	0.70057	0.70057	76.17	0
Square	3	2.93903	0.97968	106.52	0
Z_1_^2^	1	1.78433	1.78433	194.01	0
Z_2_^2^	1	0.4082	0.4082	44.38	0
Z_3_^2^	1	0.33607	0.33607	36.54	0
2-Way interaction	3	0.13458	0.04486	4.88	0.033
Z_1_ Z_2_	1	0.0098	0.0098	1.07	0.332
Z_1_ Z_3_	1	0.10397	0.10397	11.3	0.01
Z_2_ Z_3_	1	0.02081	0.02081	2.26	0.171
Error	8	0.07358	0.0092		
Lack-of-fit	5	0.06691	0.01338	6.02	0.085
Pure error	3	0.00667	0.00222		
Total	19	5.30905			

*df* degrees of freedom, *SS* sum of squares, *MS* mean square

*S* = 0.0959016; *R*^2^ = 98.61%; *R*^2^_*(*adj)_ = 96.71%; *F* = MS_(Factor)_ /MS_(Error)_

where *Z*_1_ is the concentration of Na_2_HPO_4_, *Z*_2_ is the concentration KH_2_PO_4_ g/L, *Z*_3_ is the concentration of Sucrose.

The linear models of the regression analysis (*Z*_1_, *Z*_2_ and *Z*_3_) demonstrated the significance of the parameters relatively. Similarly the quadratic models *Z*_1_^2^, *Z*_2_^2^ and *Z*_3_^2^ and *Z*_1_*Z*_3_ two way interaction model were found to be significant.

#### Fit of model

The design matrix in terms of coded and real values along with the response (Biomass and PHA g/L) is represented in Table 6. The maximum biomass g/L obtained was 2.59 g/L and maximum PHA g/L was 0.88 g/L (Table 6). The competence of the CCD model and the fitness evaluation was performed by ANOVA and the statistical significance of the model was predicted based on the *F* test and *P* test for analysis of variance (ANOVA) is given in Tables 7 and 8. The model *F* value of 51.75 (Biomass g/L in Table 7) and 7.95 (PHA g/L in Table 8) indicates the significance of the model and the lack of fit value of 0.0133 and 0.79 for biomass and PHA respectively was found to be less than the *F* values confirming the significance of the model in predicting the overall PHA yield with respect to biomass.

**Table 8:** Analysis of variance for PHA (g/L) with three factors

Source	*df*	Adj SS	Adj MS	*F* value	*p* value
Model	11	0.750316	0.068211	7.95	0.003
Blocks	2	0.041597	0.020799	2.42	0.15
Linear	3	0.216226	0.072075	8.4	0.007
Z_1_	1	0.119025	0.119025	13.87	0.006
Z_2_	1	0.021025	0.021025	2.45	0.156
Z_3_	1	0.076176	0.076176	8.88	0.018
Square	3	0.431045	0.143682	16.74	0.001
Z_1_^2^	1	0.056745	0.056745	6.61	0.033
Z_2_^2^	1	0.050508	0.050508	5.89	0.041
Z_3_^2^	1	0.21603	0.21603	25.17	0.001
2-Way interaction	3	0.057634	0.019211	2.24	0.161
Z_1_ Z_2_	1	0.041472	0.041472	4.83	0.059
Z_1_ Z_3_	1	0.0128	0.0128	1.49	0.257
Z_2_ Z_3_	1	0.003362	0.003362	0.39	0.549
Error	8	0.068657	0.008582		
Lack-of-fit	5	0.039085	0.007817	0.79	0.618
Pure error	3	0.029572	0.009857		
Total	19	0.818973			

*df* degrees of freedom, *SS* sum of squares, *MS* mean square

*S* = 0.0926396; *R*^2^ = 91.62%; *R*^*2*^_(adj)_ = 80.09%; *F* = MS_(Factor)_ /MS_(Error)_

#### Interaction of variables on responses

**Fig. 3:**
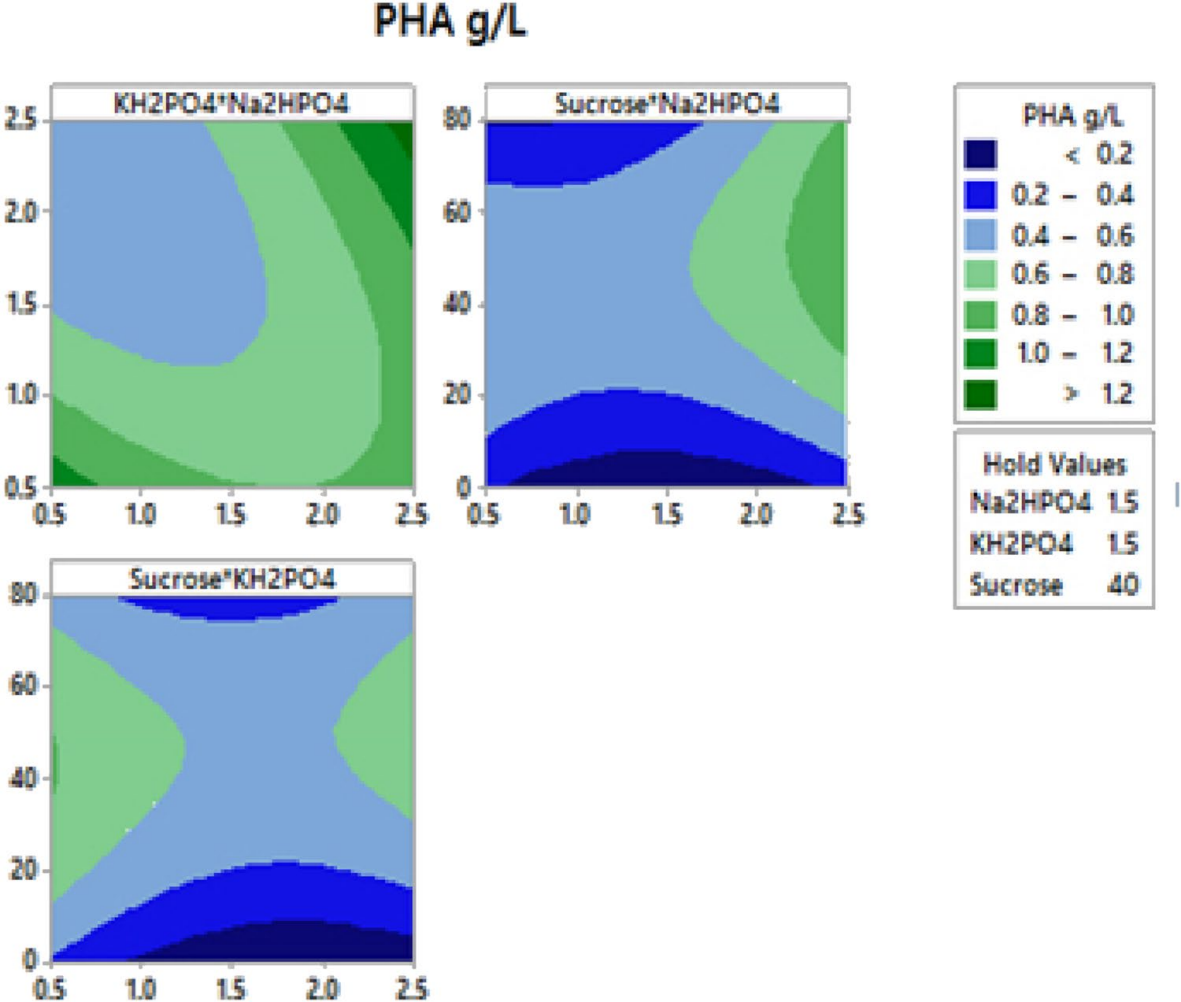
2D contour plot showing the interactive effects of three variables and on PHA production by *B.endophyticus* using sucrose as carbon source

The two dimensional (2D) contour plot are usually used to visually represent the regression equations to study the interaction effect of independent and dependent variable. In these plots, two response factors are presented keeping other factor at constant and the extent of interaction is usually determined by the shape of the plot. To study the interaction effect between three factors that is significantly affecting biomass and PHA accumulation was well studied using 2D plots as represented in Figs. 2 and 3 respectively. The circular contour plot Fig. 2a between KH_2_PO_4_ and Na_2_HPO_4_ shows that there is only less interaction between these factors in increasing biomass accumulation.

The factorial design enabled to restrict the number of experiment to 15 and gave an idea about the higher order interaction among the factors. All the experiments were conducted in a randomized manner. The synergistic effect of 2 or more factors could be thoroughly analyzed. These significant parameters were taken up for optimization using central composite design. For further confirmation and to obtain the right combination, those factors which showed influence was taken for central composite design and helped in concluding the right combination of parametrs. The coefficient of determination *R*^2^ value determines fit of the model which was 0.9162 (*p* < 0.05), 91.62% for PHA and 0.9861 (*p* < 0.05), 98.61% for biomass production. This enables in identifying the variability of the response which can be predicted. The observed model was relevant in predicting the optimal growth and PHA biosynthesis in *B. endophyticus* using sucrose as carbon source based on the predicted values given by the statistical CCD model.

The elliptical shape of contour plot as in Fig. 2a indicates a negative effect on PHA production with respect to higher concentration of KH_2_PO_4_ and Na_2_HPO_4_ which decreases PHA production. This was in well agreement with the necessity of nutrient limiting condition for organism to initiate PHA production. In our another study, it was reported that PHA production in the presence of inexpensive carbon source like sugarcane molasses (4%) with increased agitation rate enhanced PHA production by 10.7 g/L with 15.37 g/L of cell mass statistically. This experimental model created using CCD reported that increase in sugar concentration (> 4%) had a negative influence on biopolymer production (Geethu et al. 2019).

Mohanrasu (2020) reported the importance of nitrogen, potassium and sodium concentration in the basal medium for cell growth for the PHA producers which later on limiting the nutrients enhance the production. 2.74 g/L of PHA was reported by the same authors in *B. megaterium* strain utilizing glucose as carbon source (Mohanrasu et al. 2020). Similarly in this study as well, the significance and dependency of the strain on N, P and Na source were clearly shown as a prompt requirement to increase the cell mass. Later for PHA production, limitation of the nitrogen source was found to be significant which shows the limitation of nutrients except carbon enhances PHA production (Narayanan et al. 2021). The conclusive finding of the study is that the basal medium can be enriched with essential nutrients for fast cell growth and later during the late log phase reducing the nitrogen source can amplify PHA production. The insignificant correlation obtained during statistical optimization can be correlated in this case. Similar results were reported by another group where in growth of *B. aryabhattai* were found to excess in nutrient rich medium given initially with extra nitrogen source which on depletion of the same enhanced PHA production (Pillai et al. 2017). Another study reported that PHB was cell-growth-associated and PHA production started with log phase around 36 h and increase to maximum around 48 h with minimal nitrogen sources (Yao et al. 2012).

### Bioreactor studies for the optimised medium

PHA production in the excess presence of sucrose by *B. endopyticus* was further scaled up in 3 L bioreactor (New Brunswick Scientific, Bioflo 110) using optimal media components suggested by statistical model. A working volume of 1 L of production media with optimal concentration of KH_2_PO_4_, Na_2_HPO_4_ and sucrose with 1.5, 1.5 g/L and 4% was used respectively for batch cultivation. As per statistical analysis (CCD) the obtained PHA % obtained using this optimal condition was 46.5 (11th run Table 6). 250 rpm agitation rate with the desired dissolved oxygen of 100% was maintained during 72 h of batch cultivation. In this scale up study, it was found that at 72 h the PHA production was 49.9% which was even higher than the production obtained during the statistical experimental run. So this optimised media was in well accordance with the suggested model and can be used further for increased production. A similar statistical study was conducted previously under controlled medium condition with initial 4% sugar content provided by SCM at 250 rpm by test organism which gave upto 68.5% of PHA. This result was published and was also found that PHA accumulation started during the late log phase utilising the sugar content as expected and gave a maximum production of 5.85 g/L from 8.53 g/L of biomass at 72 h (Geethu et al. 2019).

**Fig. 4:**
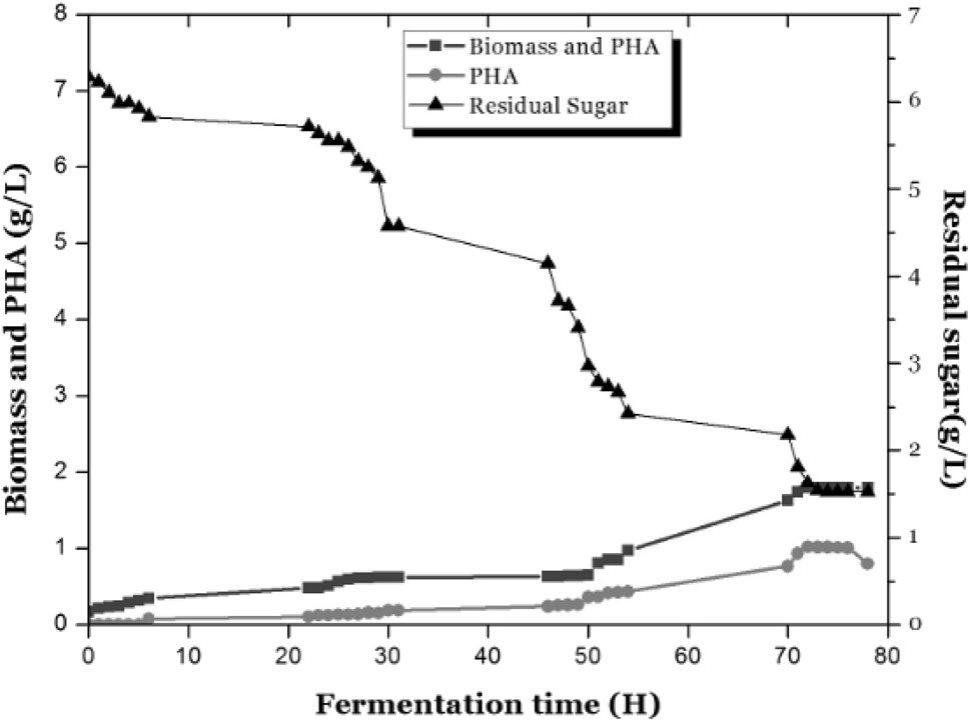
Growth of *B. endophyticus* with 1 L optimal production media in a 3 L bioreactor PHA

The 1 h interval time course growth study of *B. endophyticus* during growth in the bioreactor revealed that the organism directly utilise sucrose and produce PHA during the growth phase (Fig. 4). Biomass was increasing constantly till the nutrients got exhausted till 72 h and about 1.72 g/L of biomass and 0.8 g/L of PHA (49.47% of PHA) was gravimetrically analysed during cultivation and started to reduce after 72 h. The optimal production rate was observed during 68–72 h, indicating that late log phase is the ambient culturing time to extract the polymer before getting utilised by the same strain owing to favourable conditions that may prevail (Fig. 4). The production was found to be reduced as part of the reutilisation by the organism which gave only 38.81% of PHA. Based on the result depicted in Fig. 4, 70–72 h were considered to be optimum for the maximum production of PHA in *B. endophyticus,* beyond which the production terminates due to sporulation and cessation of cell growth due to intracellular utilisation of PHA (Valappil et al. 2007; Nair et al. 2014). Application of different statistical design enables proper planning to study the interactions between different parameters. Difference in the PHA production rate is mainly due to the substrate variability and variability of parameter during the production phase (Mohanrasu et al. 2020). The efficient interaction of various parameters with minimal experimental design enables to generate maximal process response.

Based on the result, 70–72 h, late stationary phase was considered to be optimum for the maximum production of PHA in *B. endophyticus,* beyond which the production terminates due to sporulation and cessation of cell growth, or due to intracellular utilisation of PHA. PHA seems to get reduced after 76 h due to reutilisation of the energy storage and hydrolysis reaching stationary phase wich suggests the critical time to harvest the biomass for the optimal gain of PHA. In the low sugar environment provided by the medium during late stationary phase, or in the sugar depleting condition, hydrolysis happens which is mediated by enzymes especially produced by *PhaZ* gene within the strain (Mansfield et al. 1995; de Andrade Rodrigues et al. 2000). The culture pH tend to decrease from initial pH of 7.2–4.8, which inturn effects the cell growth during the stationary stage. But in this condition, the accumulation of PHA can be assumed to be maximum in the existing cells due to the harsh conditions provided in the stationary phase. The main fact is that PHA acts as the carbon source leading to the reduction in the cytoplasmic PHA during the late phases of production itself. The specific optimal production phase of the strain was found to be during late log phase/stationary phase. So any stratergies that can prolong growth phase of the organism enable to increase the PHA yield per biomass.

### ***FTIR and ***^***1***^***HNMR analysis of the obtained polymer***

**Fig. 5:**
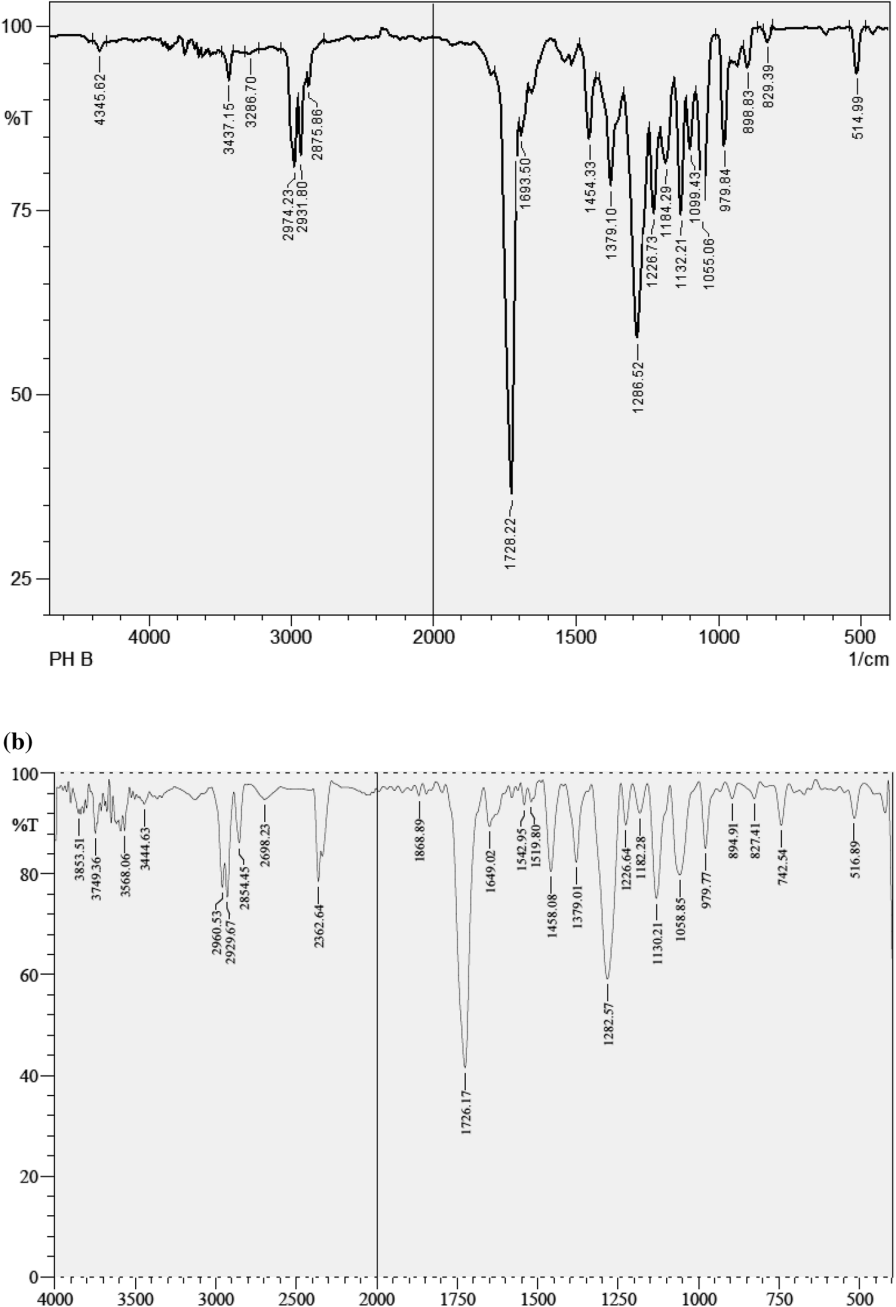
FTIR spectra of **a** standard PHB and **b** PHA extracted from *B. endophyticus* using 4

Characterisation of extracted PHA film was confirmed by Fourier transform Infrared (FTIR) and ^1^H nuclear magnetic resonance (NMR) spectroscopy (Kulkarni et al. 2011). Characterization of PHA obtained from *B. endophyticus* was represented in Fig. 5b with a characteristic peak C=O stretch at 1726 cm^−1^ corresponding to the ester group. The strong adsorption band around 1379, 1458, 2929, 1649 and 3749 cm^−1^ corresponds to –CH_3_, –CH_2_, CH, C–O, and O–H groups respectively, which was reported to be similar with pure PHB (Fig. 5a).

**Fig. 6:**
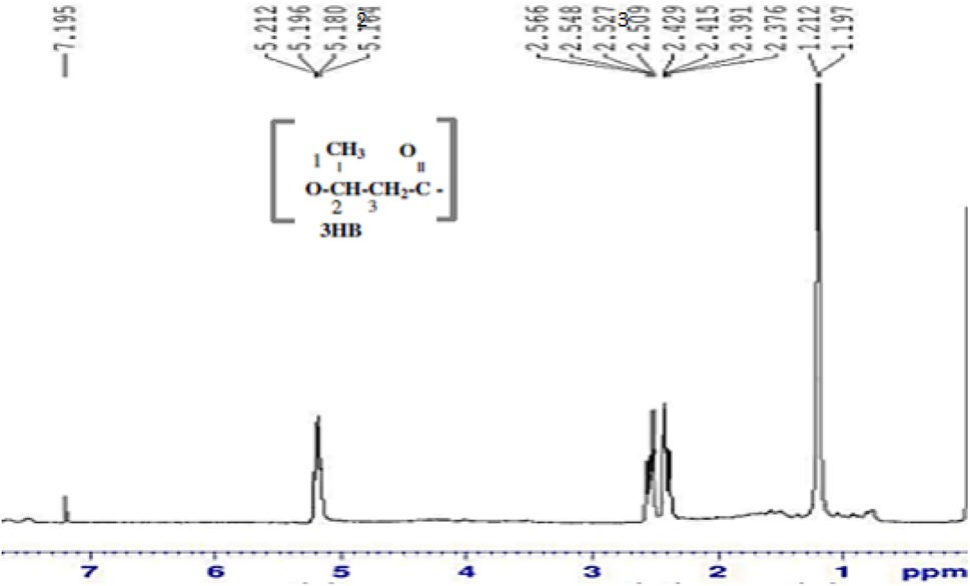
^1^H NMR spectra of PHA extracted from *B. endophyticus* using 4% sucrose as carbon source

^1^H NMR is routinely used as an analytical technique to determine PHA produced. In this study, PHA film obtained after each trial was characterised by ^1^H NMR. Similar spectral pattern with the standard PHB spectra was obtained (Fig. 6) (Chaijamrus and Udpuay 2008). Characteristic doublet at 1.299 ppm indicated the presence of methyl group was found coupled with single proton. The peak at 2.57 ppm, Doublet of quadruplet at corresponding methylene group adjacent to an asymmetric carbon bearing single atom was observed. The methylene group characteristic Multiplet was found precisely on 5.27 was determined from the ^1^H NMR spectra (Narayanan et al. 2021).

## Conclusing remarks

The process development stratergy for *B. endophyticus,* an aerobic PHA producer mainly relay on two basic principles like oxygen utilisation behaviour and its capability to cope up with the fermentation parameters economically to synthesise PHA. Experimental studies were conducted to identify and optimise factors that significantly influence PHA production in bioreactor. The different parameters influencing the biomass accumulation which in turn increases the PHA synthesis has to be identified and optimized for maximum PHA production. For this, conventional optimization process was carried through bioreactor. PHA producing *B. endophyticus* was found to be an excellent strain to utilize simple sugars like sucrose was an outcome of this study. This study has led to the conclusion in correlating the biomass and PHA production by optimizing two different media conditions so as to increase cell mass during the initial stages and PHA production in the latter stages by altering the media ingredients. The optimization studies showcased the requirement of potassium, and sodium requirement of the strain for growth and maintenance of cell, whereas deficiency of nitrogen source increased PHA production. PHB of 49% was obtained from the lab scale bioreactor which was higher than any other PHB producing *Bacillus s*train utilizing sucrose as sole source of carbon. Further cheap feed stocks rich in sucrose content can be replaced to reduce the overall production rate of PHB in large scale for industrial production.

## Abbreviations

PHA: Polyhydroxyalkanoate
CCD: Central composite design
PB: Plackett–Burman design
RSM: Response surface methodology
PM: Production medium

## Declarations

### Availability of data and materials

It will be provided on request and there is no supplementary data provided.
